# Role of gut microbiota in melanosis coli: from anthraquinone biotransformation to mucosal homeostasis dysbiosis

**DOI:** 10.3389/fphar.2026.1791164

**Published:** 2026-06-17

**Authors:** Peng Zhang, Yuan-dan Zhuang, Wen-wen Lv, Yuan Zhao, Ji-hou Wang, Jv-ying Zhang, Li-long Wu

**Affiliations:** 1 Qujing Hospital of Traditional Chinese Medicine, Qujing, Yunnan, China; 2 Mengzi Hospital of Traditional Chinese Medicine, Mengzi, Yunnan, China

**Keywords:** anthraquinone laxatives, biotransformation, dysbiosis, gut microbiota, intestinal barrier, melanosis coli, mucosal homeostasis, narrative review

## Abstract

Melanosis coli (MC) is a benign and usually reversible condition characterized by brownish-black pigmentation of the colonic mucosa and is commonly associated with chronic exposure to anthraquinone laxatives (ALs). The best-established histopathological sequence involves AL-related epithelial apoptosis, phagocytosis of apoptotic bodies by macrophages, and subsequent lipofuscin deposition. Emerging evidence suggests that the gut microbiota (GM) may contribute to this process by converting pharmacologically inactive anthraquinone glycosides into active anthrone metabolites, including rhein anthrone. This narrative review summarizes available MC-specific findings and clearly distinguishes them from mechanistic hypotheses extrapolated from constipation, intestinal barrier, and microbiome literature. We discuss microbial β-glucosidases and reductases involved in AL biotransformation, reported changes in microbial diversity and SCFA-producing taxa in MC or constipation-associated cohorts, and plausible links with barrier dysfunction, bile-acid metabolism, tryptophan-derived metabolites, and LPS-TLR4 signaling. We therefore present the “Microbiota-Apoptosis Axis” as a proposed framework rather than a validated causal pathway. Finally, we review GM-targeted strategies, including probiotics, synbiotics, and fecal microbiota transplantation, while emphasizing that direct clinical evidence in MC remains limited and that cessation of anthraquinone laxatives remains the primary management strategy.

## 1 Introduction

Melanosis coli (MC) is a benign condition commonly found during routine colonoscopy that is characterized by dark brown or black discoloration of the colonic mucosa. MC is histologically defined by the accumulation of lipofuscin-laden macrophages within the lamina propria of the colon (Benavides et al., 1997; Bechara et al., 2016). MC is an indicator of chronic intestinal dysfunction and the long-term use of anthraquinone laxatives (ALs) (Zhang R. et al., 2023; Bai et al., 2025). The association between MC and colorectal cancer risk remains a subject of debate. Available meta-analytic and observational evidence suggests that MC may be associated with increased detection of hyperplastic polyps or adenomas, while a consistent increase in colorectal cancer risk has not been established; therefore, clinical vigilance should be based on the patient’s overall colorectal risk profile rather than on MC alone (Katsumata et al., 2021; Lombardi et al., 2022; Zhong and Liu, 2025).

ALs are derived from natural sources such as Rhei Radix et Rhizome (rhubarb), aloe, and senna, and are widely used over-the-counter treatments for chronic constipation (Yang et al., 2020). Epidemiological studies demonstrate that >90% of MC cases are associated with the prolonged consumption of these compounds (Liu et al., 2017). The prevailing theory of MC pathogenesis suggests that active AL metabolites can induce apoptosis in colonic epithelial cells. These apoptotic bodies are then engulfed by resident macrophages, which accumulate lipofuscin, a byproduct of cellular degradation responsible for the dark pigmentation (Walker et al., 1988; Lee et al., 2016).

The gut microbiota (GM) is likely to play an important permissive and modulatory role in AL-associated MC, although direct causal evidence in MC remains incomplete. ALs, such as sennosides, are large hydrophilic glycosides that are pharmacologically inactive in their native form. They pass largely unabsorbed through the stomach and small intestine and undergo extensive biotransformation in the colon (Wilson and Nicholson, 2017; Le et al., 2021). Their activation into purgative and potentially cytotoxic metabolites depends substantially on enzymatic activity within the colonic microbiota (Pant et al., 2023; Xu et al., 2025).

Furthermore, chronic AL exposure and constipation-associated ecological changes have been associated with dysbiosis, which may contribute to mucosal vulnerability and persistence of bowel dysfunction (Zhang R. et al., 2023; Mishra et al., 2025). This review aims to bridge the gap between the pharmacological and microecological understanding of MC by analyzing the tripartite relationship between ALs, the GM, and MC pathogenesis. Specifically, this review focuses on (1) mechanisms by which the GM activates ALs; (2) characteristics of dysbiosis and its potential impact on intestinal barrier function; (3) plausible molecular pathways linking dysbiosis and apoptosis; and (4) the current and future clinical potential of GM-targeted interventions. By integrating these perspectives, we propose a testable, hypothesis-generating model that views MC as a mucosal response to microbiota-mediated metabolism of ALs and host barrier susceptibility.

### 1.1 Review type and literature search strategy

This article is a narrative review with a mechanistic focus rather than a systematic review or meta-analysis. To improve transparency, we performed a structured literature search in PubMed/MEDLINE, Web of Science, Scopus, and Google Scholar for publications available up to January 2026. Search terms included combinations of “melanosis coli,” “anthraquinone laxatives,” “senna,” “sennoside,” “rhein anthrone,” “rhubarb,” “aloe,” “gut microbiota,” “microbiome,” “dysbiosis,” “β-glucosidase,” “reductase,” “short-chain fatty acids,” “intestinal barrier,” “TLR4,” “probiotics,” “synbiotics,” and “fecal microbiota transplantation.” Reference lists of relevant reviews and original articles were also screened manually.

We prioritized human MC cohorts, case-control studies, meta-analyses, and experimental studies directly addressing anthraquinone biotransformation. Because MC-specific microbiome studies remain limited, we also included mechanistic studies from chronic constipation, intestinal barrier dysfunction, and general microbiome research when they were directly relevant to the proposed pathways. Such extrapolated evidence is explicitly identified throughout the manuscript. Studies were excluded when they did not address MC, anthraquinone metabolism, microbiota composition/function, intestinal barrier mechanisms, or clinical interventions relevant to MC.

## 2 Gut microbiota: the engine of anthraquinone activation

The pharmacological activity of ALs is intrinsically linked with the metabolic capacity of the colonic microbiota. ALs, such as sennosides, are large O-glycosides that are too polar to be absorbed in the upper gastrointestinal tract (Yang et al., 2020). They function as prodrugs and require enzymatic cleavage and reduction by intestinal bacteria to release active metabolites in the colon, where they exert laxative effects and may contribute to melanosis-inducing epithelial injury (Le et al., 2021; Zhang M. M. et al., 2023). This process illustrates how the gut microbiome can function as an extrahepatic drug-metabolizing system (Wilson and Nicholson, 2017; Pant et al., 2023).

The bacterial taxa and enzymatic functions involved in AL activation and mucosal protection are summarized in Table 1. Importantly, the same genus may contain strains with divergent functions; therefore, therapeutic interpretation should be strain-specific rather than genus-wide.

**TABLE 1:** Key bacterial taxa and functional pathways implicated in anthraquinone biotransformation and melanosis coli pathogenesis.

Bacterial genus/Species or functional group	Evidence context	Role in MC pathogenesis	Key enzyme/Metabolite/Pathway	Interpretation and host impact
Bifidobacterium (e.g., Bifidobacterium sp. strain SEN; B. pseudocatenulatum)	Direct anthraquinone metabolism evidence; strain-level differences	Hydrolysis of ALs and, in selected strains, reductive activation	β-glucosidase; reductase/nitroreductase activity	Can release anthraquinone aglycones and contribute to rhein anthrone formation; therapeutic use requires strain screening rather than genus-level assumptions.
Lactobacillus	General anthraquinone hydrolysis and probiotic literature	Possible hydrolysis of ALs; potential barrier support in selected strains	β-glucosidase; SCFA- and barrier-associated effects in some strains	May be beneficial or metabolically activating depending on strain; should be selected based on functional testing.
Bacteroides and Clostridium	Experimental and inferred anthraquinone reduction evidence	Reduction of aglycones to active anthrones	Reductases and anaerobic electron-transfer reactions	May contribute to toxic rhein anthrone generation; relevance should be validated in MC cohorts.
Sennoside A-reducing guilds (e.g., StNfrA-positive taxa)	Recent enzyme-activity visualization studies	Functional activation of sennoside A	StNfrA-like nitroreductase; sennoside a reductase	Potential biomarker of anthrone-activating capacity; not yet validated clinically in MC.
Faecalibacterium and roseburia	MC/constipation-associated depletion; barrier literature	Mucosal protection	Butyrate and other SCFAs	Support colonocyte energy metabolism, tight junctions, and anti-inflammatory tone; depletion may increase vulnerability to injury.
Escherichia-Shigella and other gram-negative taxa	Reported enrichment in MC or constipation-associated dysbiosis	Potential pro-inflammatory amplification	LPS-TLR4/NF-κB signaling	May contribute to inflammatory priming and epithelial susceptibility; causality in MC remains unproven.
Bile-acid- and tryptophan-metabolizing taxa	Mechanistic extrapolation from intestinal barrier/microbiome literature	Potential alternative pathways beyond SCFAs	Secondary bile acids; indole derivatives; FXR/TGR5 and AhR signaling	Important candidate mechanisms for future MC-specific multi-omics research.

### 2.1 Two-step biotransformation pathway mediated by gut microbiota

**FIGURE 1:**
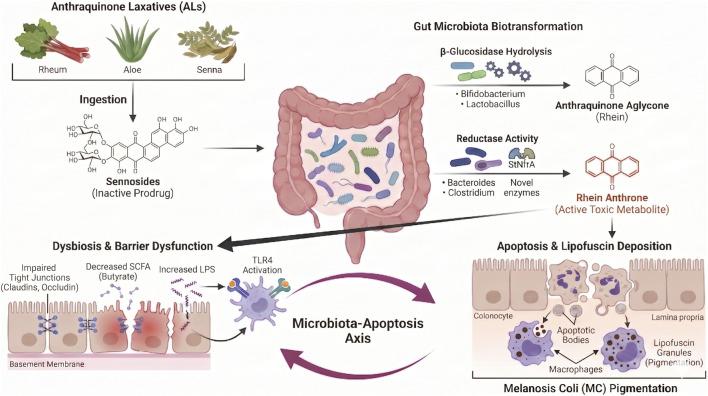
The Microbiota-Apoptosis Axis in Melanosis Coli Pathogenesis. Proposed Microbiota-Apoptosis Axis in anthraquinone-associated melanosis coli. Anthraquinone glycosides such as sennosides reach the colon largely unchanged and are converted by microbial β-glucosidases into anthraquinone aglycones, followed by reductase/nitroreductase-mediated conversion to rhein anthrone. The figure indicates that rhein anthrone, rather than the parent sennosides alone, is the downstream metabolite most directly linked to epithelial secretion, cytotoxicity, and apoptosis. Chronic AL exposure and constipation-associated dysbiosis may reduce SCFA-producing taxa and barrier-supportive metabolites, alter bile-acid and tryptophan-derived signaling, increase luminal LPS, and activate TLR4/NF-κB signaling in epithelial cells and lamina propria macrophages. The immune cells illustrated in the lamina propria are macrophages that phagocytose apoptotic bodies and accumulate lipofuscin granules, producing the characteristic MC pigmentation. Solid arrows indicate supported metabolic or histopathological steps; dashed arrows indicate hypothesized dysbiosis-barrier-immune links requiring MC-specific validation.

The conversion of inactive AL glycosides into active anthrones involves a two-step biotransformation process that is sequentially mediated by the GM (Huang et al., 2019) (Figure 1).

#### 2.1.1 Step 1: hydrolysis (deglycosylation)

The initial rate-limiting step involves hydrolysis of the glycosidic bonds. ALs are first cleaved by β-glucosidases secreted by specific colonic bacteria, leading to release of the sugar moiety and formation of corresponding anthraquinone aglycones, such as rhein (Cui et al., 2019). Bifidobacterium and *Lactobacillus* include strains capable of participating in this deglycosylation process, although β-glucosidase activity is strain-dependent and should not be generalized to all members of these genera. Specific strains of Bifidobacterium, such as Bifidobacterium sp. strain SEN, possess highly efficient β-glucosidase activity capable of hydrolyzing sennosides (Akao et al., 1994).

#### 2.1.2 Step 2: reduction

The anthraquinone aglycones are then further reduced by anaerobic bacteria to form pharmacologically active metabolites such as rhein anthrone (Kon et al., 2014). This reduction is a critical step because the anthrone product is more biologically active and potentially more cytotoxic than the aglycone. Recent studies have identified specific enzymes and bacteria involved in this process. Zhai et al. developed an enzymatic activity visualization platform to identify functional guilds of sennoside A-reducing bacteria *in situ* and discovered a novel sennoside A-reducing enzyme, StNfrA, a type of nitroreductase (Zhai et al., 2025). Previous studies have identified genera such as *Bacteroides* and *Clostridium* as possible contributors to this process (Kon et al., 2014). Xu et al. used photoaffinity probes to investigate sennoside A reductase and confirmed that transformation into rhein anthrone can be catalyzed by a sequence of gut microbial hydrolases and reductases (Xu et al., 2025).

Rhein anthrone is a reactive compound that can contact and be absorbed by the colonic mucosa and is responsible for important laxative effects. Rhein anthrone reduces water and electrolyte absorption from the colon into the bloodstream, thereby increasing stool fluid content; moreover, it stimulates colonic motility, leading to the purgative effect (Wei et al., 2020; Zhang M. M. et al., 2023). Experimental and histopathological evidence supports that anthraquinone metabolites, including rhein anthrone, can induce epithelial apoptosis, thereby providing a plausible trigger for lipofuscin deposition and MC (Walker et al., 1988).

The efficiency of this biotransformation pathway is likely to influence both laxative efficacy and susceptibility to AL-associated mucosal injury. Interindividual variation in microbial composition and enzymatic activity may partly explain why only some long-term AL users develop clinically evident MC (Zhang R. et al., 2023).

## 3 The landscape of gut dysbiosis and impaired barrier function in MC

ALs and their active metabolites may exert selective pressure on the composition and function of the microbial community. However, available evidence does not yet establish whether dysbiosis is a cause, consequence, or correlate of MC, because constipation, diet, age, comorbidities, and laxative exposure can all influence GM structure (Zhang R. et al., 2023; Mishra et al., 2025).

Evidence boundary. In this section, findings directly reported in MC cohorts are distinguished from mechanisms inferred from chronic constipation, intestinal barrier, and general microbiome studies. The latter are included to formulate testable pathways but should not be interpreted as MC-specific proof.

### 3.1 Alterations in microbial diversity and composition

Available clinical studies suggest altered gut microbial profiles in MC patients compared with controls, but the number of MC-specific cohorts remains small and methods differ across 16S rRNA gene sequencing and metagenomic studies.

#### 3.1.1 Reduced diversity and loss of beneficial taxa

MC patients often exhibit reduced α-diversity (species richness and evenness) in available reports (Liu et al., 2017), and similar reductions are frequently reported in chronic constipation cohorts. These changes are frequently accompanied by a decrease in beneficial bacteria, especially short-chain fatty acid (SCFA) producers (Holscher, 2017). SCFA-producing genera, such as Faecalibacterium (e.g., F. prausnitzii) and Roseburia, are crucial for maintaining colon health (Nie et al., 2021; Mohebali et al., 2023). Their primary metabolite, butyrate, is the main energy source for colonocytes and is required for maintaining epithelial barrier integrity, regulating immune responses, and influencing cellular processes through its action as a histone deacetylase inhibitor (Hamer et al., 2008; Martinez-Ruiz et al., 2025; Yang et al., 2025). Depletion of these taxa may compromise mucosal homeostasis and could increase vulnerability of the colon to anthrone-associated injury, although this inference requires MC-specific functional validation (Herrnreiter et al., 2025).

#### 3.1.2 Increase in pro-inflammatory and pathogenic taxa

MC patients have been reported to show increased abundances of potentially pro-inflammatory or pathogenic bacteria, including certain Escherichia-Shigella taxa (Liu et al., 2017). This pro-inflammatory shift may reflect enrichment of Gram-negative organisms and may contribute to low-grade mucosal inflammation, but independence from constipation severity, bowel preparation, and laxative exposure has not been definitively demonstrated (Li et al., 2019).

### 3.2 Functional dysbiosis: SCFAs, barrier function, and additional metabolic pathways

A plausible functional consequence of this taxonomic shift is reduced microbial production of SCFAs, especially butyrate (Herrnreiter et al., 2025). The lack of sufficient butyrate can impair colonocyte energy metabolism and weaken the intestinal barrier, a phenomenon often discussed as increased intestinal permeability or “leaky gut” (Marcari et al., 2025). The intestinal epithelial barrier is a complex, multi-layered system that selectively regulates the passage of molecules from the gut lumen into the bloodstream (Barbara et al., 2021; Neurath et al., 2025). The integrity of the intestinal epithelial barrier is maintained by tight junctions, including claudins, occludin, and zonulin-associated pathways, which are increasingly recognized as biomarkers for intestinal permeability (Gorecka et al., 2024; Hu et al., 2025).

A butyrate-deficient environment can lead to downregulation of tight junction proteins and increased intestinal permeability. If present in MC, compromised barrier function could increase mucosal exposure to rhein anthrone and microbial products such as lipopolysaccharide (LPS), thereby plausibly lowering the threshold for epithelial apoptosis and subsequent lipofuscin deposition (Kon et al., 2014; Skinner et al., 2025).

Beyond SCFAs, bile-acid metabolism and tryptophan-derived microbial metabolites may also be relevant to MC. Bile acids regulate epithelial secretion, motility, antimicrobial tone, and farnesoid X receptor/TGR5 signaling, whereas indole derivatives from tryptophan metabolism can modulate aryl hydrocarbon receptor pathways and mucosal barrier repair. These pathways have not yet been adequately characterized in MC cohorts, but their inclusion broadens the mechanistic model beyond a solely SCFA-centered explanation.

## 4 Molecular mechanisms: a proposed synergistic microbiota-apoptosis axis

We use the term “Microbiota-Apoptosis Axis” as a proposed conceptual framework rather than as an experimentally validated pathway. This framework hypothesizes that anthrone toxicity, microbial dysbiosis, barrier impairment, and mucosal immune activation may converge to promote epithelial apoptosis and lipofuscin deposition in MC.

### 4.1 Synergistic cytotoxicity via LPS-TLR4 signaling

Rhein anthrone, the active metabolite, is cytotoxic and has been linked to inhibition of key transport processes such as Na+/K + -ATPase activity and to mitochondrial dysfunction in colonocytes (Walker et al., 1988). Gut dysbiosis may amplify this damage. Dysbiosis characterized by increased Gram-negative bacteria can increase luminal or circulating LPS, a potent pro-inflammatory endotoxin (Cani et al., 2008; Li et al., 2019). LPS activates Toll-like receptor 4 (TLR4) signaling in colonocytes and immune cells (Chen et al., 2024; Wan et al., 2024), leading to downstream NF-κB pathway activation and release of pro-inflammatory cytokines such as tumor necrosis factor-alpha (TNF-α) (Kon et al., 2014; Wei et al., 2025). On this basis, we hypothesize that a microbiota-LPS-TLR4 inflammatory milieu could lower the apoptotic threshold of epithelial cells and increase susceptibility to rhein anthrone. This proposed synergy remains to be confirmed in MC-specific organoid, animal, and prospective human studies (Hamer et al., 2008; Lee et al., 2016).

### 4.2 Lipofuscin formation and macrophage overload

The characteristic pigmentation of MC is caused by accumulation of lipofuscin, a lipid-protein complex, within macrophages of the lamina propria (Benavides et al., 1997; Bechara et al., 2016). Lipofuscin is non-degradable, autofluorescent material generated by incomplete degradation of phagocytosed material, especially oxidized lipids and proteins from apoptotic bodies (Terman and Brunk, 1998; Vida et al., 2017; Stillman et al., 2023). It is considered a hallmark of cellular aging and oxidative stress.

In MC, sustained epithelial apoptosis—potentially amplified by the proposed microbiota-apoptosis axis—may overwhelm the degradative capacity of resident macrophages (Walker et al., 1988). These phagocytes engulf increased numbers of apoptotic bodies but cannot fully process the cellular debris, leading to progressive accumulation of undigested, pigmented lipofuscin within macrophage lysosomes (Freeman, 2008). Subsequently, these pigment-laden macrophages aggregate in the lamina propria and manifest as macroscopic black pigmentation visible during colonoscopy (Bechara et al., 2016). The reversibility of MC is dependent on cessation of the apoptotic stimulus, particularly stopping ALs, and the subsequent slow clearance of accumulated lipofuscin over months to a year.

## 5 Clinical implications: the gut microbiota as a potential therapeutic target

Because the central causal role of the GM in MC has not yet been definitively proven, microbiota-directed approaches should currently be regarded as adjunctive or investigational rather than as replacements for the standard management strategy: cessation of anthraquinone laxatives and correction of constipation with safer long-term measures.

### 5.1 Targeted microecological interventions

#### 5.1.1 Probiotics and synbiotics

If MC patients demonstrate depletion of SCFA-producing bacteria and barrier vulnerability, targeted supplementation with probiotics or synbiotics could be explored as an adjunctive strategy to support mucosal recovery (Holscher, 2017). Probiotics, including selected Bifidobacterium and *Lactobacillus* strains, can restore microbial diversity, enhance SCFA production, and strengthen the intestinal barrier by upregulating tight junction proteins in some settings (Hamer et al., 2008; Ferris et al., 2025; Jouriani et al., 2025; Kaur, 2025). However, because some Bifidobacterium strains can activate sennosides through β-glucosidase or reductase activity, probiotic selection for MC should be strain-specific and should preferably avoid strains with high anthraquinone-activating capacity while favoring barrier-supportive and SCFA-producing phenotypes.

Recent research has investigated engineered probiotics designed to remodel the intestinal epithelial barrier, offering a glimpse into future therapeutic possibilities (Chen et al., 2025). Only limited case-level evidence is available in MC. The case reported by Zakaria et al. involved cessation of laxatives and other supportive management; therefore, improvement cannot be attributed to microbiota therapy alone (Zakaria et al., 2021). Synbiotics, which combine probiotics with prebiotics, may offer a synergistic effect and further promote a healthy gut ecosystem, but MC-specific randomized trials are lacking (Yassine et al., 2025).

#### 5.1.2 Fecal microbiota transplantation (FMT)

Fecal microbiota transplantation (FMT) should be considered experimental for MC. It may be relevant only in research settings or in carefully selected patients with severe refractory constipation and documented dysbiosis, rather than as a routine MC treatment. FMT aims to restore a healthy, diverse microbial community (Larsen and Brummer, 2024). A systematic review and meta-analysis reported symptom relief and GM remodeling in patients with chronic constipation (Wang et al., 2025). In the context of MC, FMT could theoretically reduce anthrone-activating activity, restore SCFA production and barrier function, and normalize the mucosal immune environment. These mechanisms remain speculative because direct MC-specific clinical trial data are unavailable; therefore, FMT should be discussed as a future research direction, with careful attention to donor screening, safety, and regulatory requirements.

### 5.2 Microbial biomarkers for diagnosis and prognosis

Reported microbial signatures in MC and chronic constipation provide a potential opportunity for developing non-invasive risk or recovery biomarkers, but MC-specific diagnostic performance has not yet been established (Liu et al., 2017). Ratios of anthrone-activating bacteria or enzymes, such as taxa possessing StNfrA-like reductases, to protective SCFA-producers such as Faecalibacterium could be evaluated as quantitative measures of AL-associated MC risk or severity. Furthermore, monitoring recovery of beneficial taxa and reduction of inflammatory or permeability markers could complement colonoscopy in future studies, but such biomarkers should currently be interpreted as research tools rather than validated clinical endpoints.

## 6 Conclusion and future perspectives

Melanosis coli (MC) is classically recognized as a reversible consequence of long-term anthraquinone laxative exposure, but accumulating evidence supports a broader framework in which microbial drug metabolism and mucosal susceptibility may modulate disease development and resolution. We propose a refined model in which the GM acts as an essential bio-activator that converts inert compounds into cytotoxic anthrones, whereas associated dysbiosis may compromise host mucosal defense through impaired SCFA production, altered bile-acid and tryptophan metabolism, barrier weakening, and immune activation. This model provides a plausible explanation for a synergistic cycle of apoptosis, inflammation, and lipofuscin deposition, but it remains hypothesis-generating and requires direct validation.

The future of MC research should shift from descriptive pathology to functional microecology. Key areas of future investigation include the following:

1. Enzyme and strain-level characterization: Based on recent findings (Xu et al., 2025; Zhai et al., 2025), further studies are necessary to identify the full spectrum of bacterial species and enzymes, including reductases, nitroreductases, and β-glucosidases, responsible for potent anthrone activation. This could lead to highly specific inhibitors that reduce AL activation without disrupting the wider microbial community.
2. Targeted drug delivery and safer laxative design: Future work should develop constipation therapies that avoid broad microbial activation of cytotoxic anthrones or selectively limit mucosal exposure to reactive anthrone metabolites.
3. Clinical trials on microecological interventions: Randomized controlled trials should evaluate carefully selected probiotic strains, synbiotics, dietary fiber strategies, and, where appropriate, FMT for accelerating MC reversal and improving long-term bowel function. Trials should include laxative cessation as a controlled variable.
4. Multi-omics integration: Integrated metagenomics, metabolomics, transcriptomics, and mucosal histology can identify molecular changes in the colonic mucosa and GM during the onset and reversal of MC, thereby providing a direct test of the proposed Microbiota-Apoptosis Axis.

By adopting this microecological perspective while maintaining appropriate evidentiary caution, future research can move beyond descriptive associations and develop targeted, evidence-based strategies that promote recovery of mucosal homeostasis and intestinal health.
